# Masticatory performances and maximum occlusal forces of immediate and conventional loaded two-implant supported overdentures retained by magnetic attachments: preliminary study of randomized controlled clinical trial

**DOI:** 10.1186/s40729-021-00342-x

**Published:** 2021-06-29

**Authors:** Awutsadaporn Katheng, Manabu Kanazawa, Yuriko Komagamine, Anna Miyayasu, Yoko Uehara, Daisuke Sato, Shunsuke Minakuchi

**Affiliations:** 1 Department of Gerodontology and Oral Rehabilitation, Graduate School of Medical and Dental Sciences, Tokyo Medical and Dental University, Tokyo, Japan; 2 Department of Restorative Dentistry, Faculty of Dentistry, Naresuan University, Phitsanulok, Thailand; 3 Department of Implant Dentistry, School of Dentistry, Showa University, Tokyo, Japan

**Keywords:** Immediate load, Conventional load, Masticatory performance, Overdenture, Magnetic attachment, Edentulous mandible

## Abstract

**Background:**

The appropriate loading protocol to improve masticatory performance (MP) is still unclear in elderly patients and two-implant overdentures (2-IODs) wearers. This study aimed to compare the long-term MP and maximum occlusal force of immediate loading (IL) and conventional loading (CL) of 2-IODs retained by magnetic attachments. Nineteen edentulous patients were randomly assigned to either an IL (*n*=10) or CL group (*n* = 9). In the IL group, the implant was loaded on the same day as insertion, whereas it was loaded 3 months after insertion in the CL group. Magnetic attachments were used to retain all overdentures to the implants. MP, measured by a piece of color-changeable chewing gum and a gummy jelly test, and maximum occlusal force, measured using an occlusal force measuring device, were assessed in both groups at baseline and at 3-, 4-, and 5-year follow-ups.

**Results:**

No significant differences were observed in the MP and the maximum occlusal force between the IL and CL groups at any time point. However, a significantly higher MP was observed at the 3-year time point in the IL group (*P* = 0.036). The maximum occlusal force revealed a significant correlation with MP, both with the color-changeable chewing gum and gummy jelly at 5 years.

**Conclusion:**

After long-term observation, no significant differences in MP and maximum occlusal force were observed between the IL and CL groups. However, compared to pre-implant insertion of the complete denture, the MP in the IL group significantly improved at 3 years. Furthermore, the maximum occlusal force was significantly correlated with MP at 5 years.

**Trial registration:**

UMIN, UMIN000009889. Registered on 28 January 2013.

## Background

In numerous studies, it has been observed that the masticatory performance (MP) has improved significantly in patients using mandibular overdentures and dental implants compared to patients using complete dentures (CDs) [1–5]. In the McGill Consensus Statement published in 2002, many investigators agreed that the basic restoration for an edentulous mandible should be an implant-supported overdenture with two implants placed in the anterior mandible [6]. Thus, two-implant overdentures (2-IODs) should be considered a possible alternative treatment for patients with edentulous mandibles [7, 8]. Significant improvement in MP with 2-IODs retained by a bar attachment has been reported in several studies [7, 9–12]. However, MP of 2-IODs retained by magnetic attachment was less reported than other attachments [4]. Magnetic attachments offer several advantages, including low profile, reduced horizontal stress transmission to implants, and reduced prominence of dentures. Moreover, the magnets are simple, easy to use in clinical practice, ease prosthesis insertion, and enhance patient comfort [8, 13–15]. However, the corrosion of magnet material has been identified and significantly lowered retention and stability compared to ball and locator attachments [8].

According to the conventional loading (CL) protocol, the overdenture is attached during a second procedure after a healing period of 3–6 months, with a two-stage (submerged) implant placement protocol [16–18]. For immediate loading (IL), the overdenture with attachment system is placed in occlusion with the opposing dentition immediately or in less than 48 h of implant placement, with a one-stage (non-submerged) implant placement protocol [18–21]. The use of IL protocols may offer several advantages compared to the CL protocol, including avoiding instability of the denture during the healing period, reducing multiple rounds of relining of transitional prostheses [22], shortening the treatment period [23], improving patient acceptance, improving function, and eliminating the need for a second surgical intervention [24]. On the other hand, the IL of implants may result in larger micromotions at the implant-bone interface and disturb the healing process of osseointegration [25, 26]. In a randomized controlled trial (RCT) study, Passia et al. [27] reported no significant difference in MP between the loading protocol of single implant overdentures (1-IODs) retained by ball attachment during a 4-month evaluation period. A recent systematic review with meta-analysis reported a similar implant success rate and marginal bone loss in the IL protocol compared to the CL protocol [28, 29].

Nevertheless, more robust evidence is needed to determine whether IL or CL provides satisfactory results over time for the unsplinted mandibular 2-IODs. There are no RCTs comparing the MP of mandibular 2-IODs retained by magnetic attachment with IL to that with the CL approach at the 5-year follow-up period in the current literature. Notably, these outcomes with a 3-year follow-up have been published, and only one difference was found between IL and CL in the gummy jelly test after 6 months [30]. The present RCT aimed to compare the MP and maximum occlusal force of immediately and conventionally loaded mandibular 2-IODs and compare the baseline measures within each group with those at each evaluation time point (3-, 4-, and 5-year follow-ups) after implant insertion. Secondly, we aimed to investigate the correlation between maximum occlusal force and MP at 5 years’ follow-up of 2-IODs with magnetic attachment.

## Methods

### Study design

This study was a randomized, unblinded, parallel, and controlled clinical study with a 5-year follow-up. Participants underwent oral rehabilitation with 2-IODs at the Department of Gerodontology and Oral Rehabilitation clinic in Tokyo Medical and Dental University (TMDU) between 2012 and 2013. All study participants provided written informed consent. This study was conducted in accordance with the guidelines of the Declaration of Helsinki. The recruitment and treatment protocols were conducted according to the Ethical Review Committee of the Faculty of Dentistry, TMDU (registration number 693). This study was registered with the University Hospital Medical Information Network (UMIN) Center (UMIN000009889).

### Participants

Nineteen participants were enrolled in this RCT. The inclusion criteria were completely edentulous mandible and any opposing maxillary remaining teeth conditions, edentulous mandible for at least 6 months, no bone augmentation requirement, and good oral hygiene. The exclusion criteria included the presence of any systemic conditions that included compromised implant surgery, a history of chemotherapy or radiotherapy to the head and neck region, a history of taking bisphosphonate, and heavy smoker status.

The participants were randomly assigned to either the test group (IL), where the implants were immediately loaded, or the control group (CL), where the implants were submerged in the mucosa and loaded after 3 months of healing. A preoperative prosthetic evaluation of the existing prostheses was performed by a certified prosthodontist (M. K). A randomized treatment allocation was executed using a stratified randomization method to ensure pretreatment comparability of the groups with respect to age, gender, and the American College of Prosthodontists (ACP) classification [31]. A detailed description of the randomization was described in a previous study along with the preliminary sample size calculation (*n*=10) [30].

### Surgical and prosthetic procedures

The surgical and prosthetic procedures utilized in this study followed the protocols described in a previous report [32]. The participants were required to have adequate bone volume in the intraforaminal region for placement of 2-IODs (Nobel Speedy Groovy RP, 4 mm in diameter, 10–18 mm in length; Nobel Biocare, Gothenburg, Sweden) without bone augmentation. The 2-IODs were usually positioned in the canine or lateral incisor sites. All implants were placed by the same experienced implantologist (D.S). A surgical guide was manufactured and used during surgery. Insertion torque values between 25 and 30 Ncm were considered adequate for the IL, and greater than 35 Ncm for the CL group. In the IL group, implant keepers (Magfit, Aichi Steel Co, Aichi, Japan) with a diameter of 4.7 mm and an appropriate height (3.0, 4.0, or 5.5 mm) were connected to each implant. Insertion torque values between 25 and 30 Ncm were considered adequate for the IL. Magnet assemblies were then integrated into the intaglio surface of the dentures intraorally using autopolymerizing acrylic resin (Unifast III, GC, Japan). In the CL group, two healing abutments were connected to the implants. The inner aspects of the denture base around the healing abutments were relieved during a 3-month healing period. Three months after implant surgery, the healing abutments were replaced with implant keepers of the appropriate height. Magnetic assemblies were picked up in the same manner as that described for the IL group.

### Outcome measures

MP was measured by a piece of color-changeable chewing gum and a gummy jelly test in addition to measurements of maximum occlusal force in all participants before implant placement (baseline) and at 3, 4, and 5 years after implant placement.

#### Color-changeable chewing gum

Color-changeable chewing gum (Masticatory Performance Evaluating Gum Xylitol, Lotte, Japan) was evaluated, as described by Hama et al. [33]. The participants were instructed to chew the gum 60 times freely, at a speed of one chewing cycle per second. The chewed gum was flattened to a thickness of 1.5 mm by compression in polyethylene films between two glass plates and evaluated immediately after chewing. Then, L*, a*, and b* values were measured using a colorimeter (CR-13, Konica-Minolta Sensing, Japan) using the CIECLB color system at five points: the center and approximately 3 mm above, below, to the right, and to the left of the center. ΔE values were evaluated using the following formula:

$$ \Delta  E=\sqrt{{\left({L}^{\ast }-72.3\right)}^2+{\left({a}^{\ast }+14.9\right)}^2+{\left({b}^{\ast }-33.0\right)}^2} $$

The number of chewing cycles (*N*) was calculated using the following formula and defined as the evaluation value [34].

$$ \Delta  E=73.2-\frac{2.85\times {10}^7}{1+{e}^{9.95\times {10}^{-3\left(N+1.35\times {10}^3\right)}}} $$

#### Gummy jelly

A gummy jelly (UHA Mikakuto, Japan) was used as the test item. The participants were instructed to chew the gummy jelly 30 times. Then, the patients expectorated the comminuted jelly as thoroughly as possible into a paper cup. After that, the comminuted gummy jelly was evaluated on a scale of 1–10 using the visual scoring methods described by Nokubi et al. [35].

#### Maximum occlusal force

The maximum occlusal force was measured using an occlusal force measuring device (Occlusal Force-Meter GM 10, Nagano Keiki, Japan). The devices were positioned unilaterally on the right and left sides of the first molar. Participants were instructed to bite as hard as possible on the device for 3 s. The measurements were repeated three times on each side. The average of the highest unilateral right and left measurements was used for data analysis.

### Statistical analysis

All statistical analyses were performed using SPSS software version 24 (SPSS Inc., Chicago, IL, USA). The normality of the data was tested using the Kolmogorov-Smirnov test. The baseline characteristics of the participants were compared using the non-paired *t*-test and the chi-square test. The Mann-Whitney *U* test was used to analyze differences in MP and maximum occlusal force between the two loading groups. The differences in the MP and maximum occlusal force between the baseline and 3, 4, and 5 years within each group were compared using the Wilcoxon signed-rank test. The Bonferroni correction method was applied for multiple comparisons. Pearson correlations were performed to determine the association between the maximum occlusal force and MP at the 5-year time point. Statistical significance was set at *P* < 0.05.

## Results

**Fig. 1:**
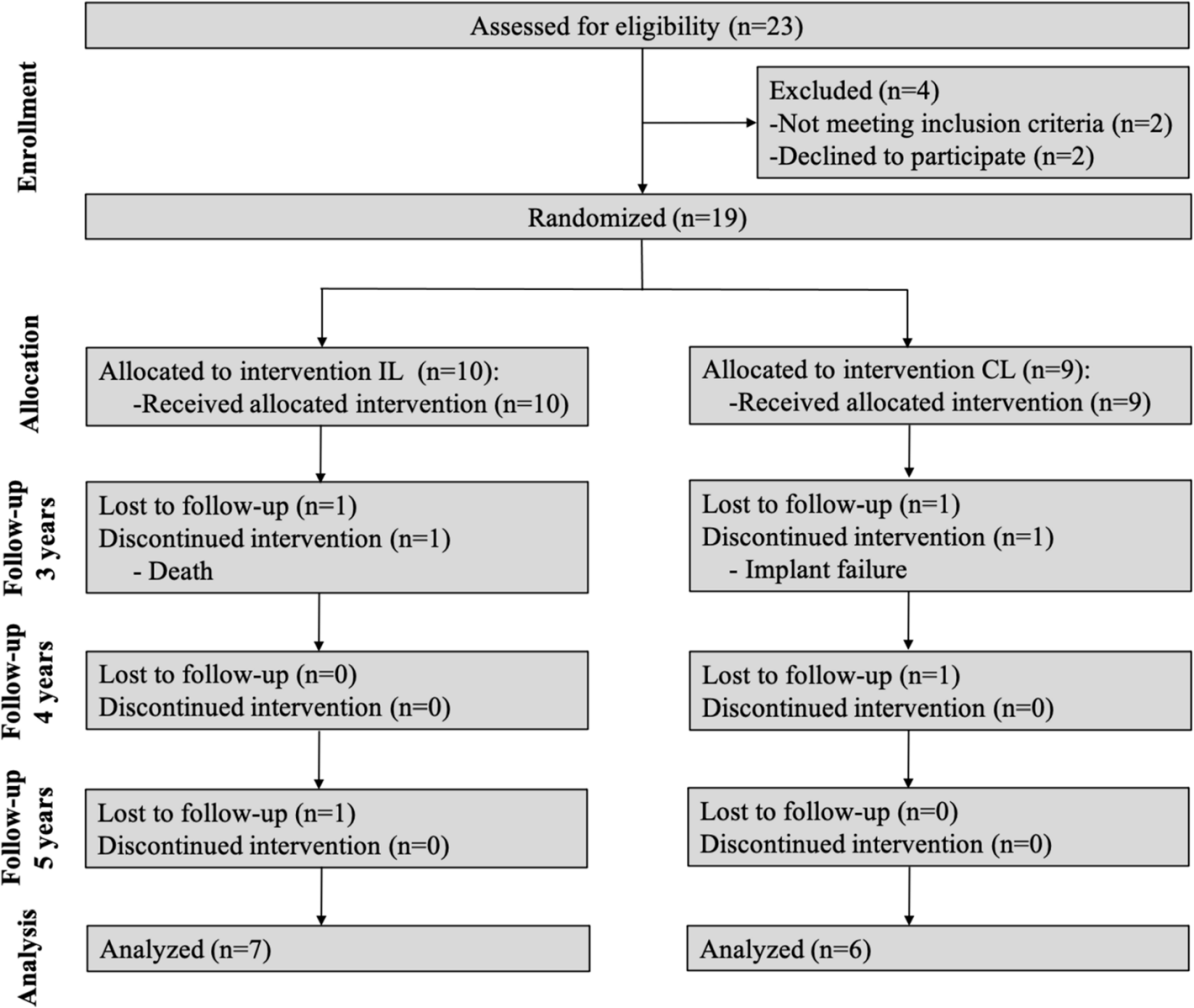
CONSORT flow diagram of participants enrollment, intervention, follow-up, and data analyses in the immediate loading group compared with the conventional loading group

The patient flow diagram and allocation during the study are reported in Fig. 1. The baseline characteristics of the participants are shown in Table 1. All the participants were wearing a CD in the maxillary arch with the exception of 3 participants in the IL group and 2 participants in the CL group. For the 3-year assessment, 15 participants were evaluated in this study. There were no significant differences in age, sex, or ACP classification between the two groups.

**Table 1:** Baseline characteristics of participants

Characteristics	Immediate group (***n***=10)	Conventional group (***n***=9)	Total (***n***=19)	***P*** value
**Age (SD)**	69.2 (10.6)	66.6 (9.1)	68.4 (9.9)	0.57^†^
**Gender**	6 men	3 men	9 men	0.25^‡^
	4 women	6 women	10 women	
**ACP classification**				0.89^‡^
**I**	2	2	4	
**II**	3	2	5	
**III**	4	3	7	
**IV**	1	2	3	
**Maxillary occlusion status**	3 dentulous	2 dentulous	5 dentulous	
	7 edentulous	7 edentulous	14 edentulous	

*SD* standard deviation, *ACP* American College of Prosthodontists

^†^Independent sample *t*-test

^‡^Chi-square test

Table 2 presents the median of MP and maximum occlusal force for 3-, 4-, and 5-year follow-ups. There were no significant differences in the color-changeable chewing gum, gummy jelly, and maximum occlusal force between the IL and CL groups at any evaluation time point. Table 3 presents the within-group comparison of MP and maximum occlusal force from baseline to 3-, 4-, and 5-year evaluation time points. The color-changeable chewing gum scores were significantly higher than the baseline at the 3-year evaluation time point in the IL group, but there were no significant differences in 4- and 5-year evaluation time points. For the CL group, there were no significant differences in any evaluation time point (Table 3). However, the color-changeable chewing gum scores in both groups tended to increase when the evaluation time point increased (Table 2). The gummy jelly score was not significantly different from baseline in any evaluation time point within each group (Table 3). The gummy jelly score in both groups tended to decrease when the evaluation time point increased (Table 2). The maximum occlusal force was not significantly different from baseline in any evaluation time point in either group (Table 3).

**Table 2:** Median values for masticatory performance and maximum occlusal force between two loading groups

	Median [first, third quartile]			
	Baseline	3 years	4 years	5 years
**Color-changeable chewing gum score**				
Immediate	60.0 [38.7, 72.7]	104.2 [94.6, 118.5]	128.9 [115.0, 144.0]	146.0 [123.8, 162.8]
Conventional	53.3 [26.4, 73.4]	110.4 [76.0, 130.2]	134.4 [121.2, 155.8]	141 [96.6, 149.3]
*P* value	0.657	1.00	0.668	0.317
**Gummy jelly score**				
Immediate	2 [0.75, 3]	5.5 [4, 6]	5 [2, 7]	3 [2, 7]
Conventional	1 [0.25, 3]	4 [1, 5]	4 [0.75, 5.25]	3.5 [2.25, 4.5]
*P* value	0.749	0.292	0.387	0.771
**Maximum occlusal force (N)**				
Immediate	165.0 [117.5, 197.8]	286.0 [254.5, 325.3]	220.0 [193.0, 445.0]	322.0 [155.0, 328.0]
Conventional	136.5 [95.8, 234.5]	268.0 [232.0, 559.0]	323.0 [153.0, 477.0]	259.0 [174.0, 397.3]
*P* value	0.824	0.728	0.886	0.886

The median, first quartile, and third quartile values for masticatory performance (color-changeable chewing gum, gummy jelly) and maximum occlusal force between two loading groups from baseline to 3, 4, and 5-year evaluation time point

**Table 3:** Comparison of *P* values in masticatory performance and maximum occlusal force within each group

	***P*** value		
	3 years	4 years	5 years
**Color-changeable chewing gum**			
Immediate	0.036*	0.054	0.054
Conventional	0.054	0.084	0.129
**Gummy jelly**			
Immediate	0.075	0.174	0.312
Conventional	0.342	0.504	0.501
**Maximum occlusal force**			
Immediate	0.075	0.189	0.054
Conventional	0.054	0.138	0.138

The *P* values for difference in masticatory performance (color-changeable chewing gum, gummy jelly) and maximum occlusal force within each group between baseline to 3, 4, and 5-year evaluation time point

*P value < 0.05

**Fig. 2:**
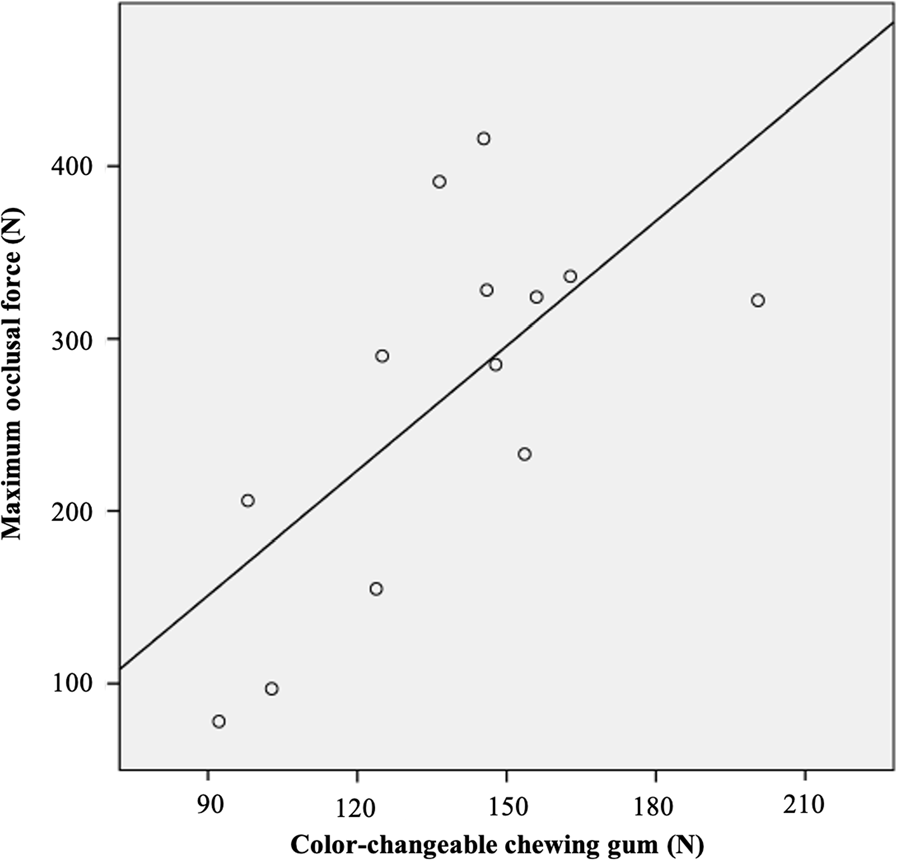
The scatter diagram shows the relationship between maximum occlusal force and color-changeable chewing gum. Significant positive correlation was observed (*r* = 0.676, *P* = 0.007) in a 5-year follow-up period. Solid line represents the regression line

**Fig. 3:**
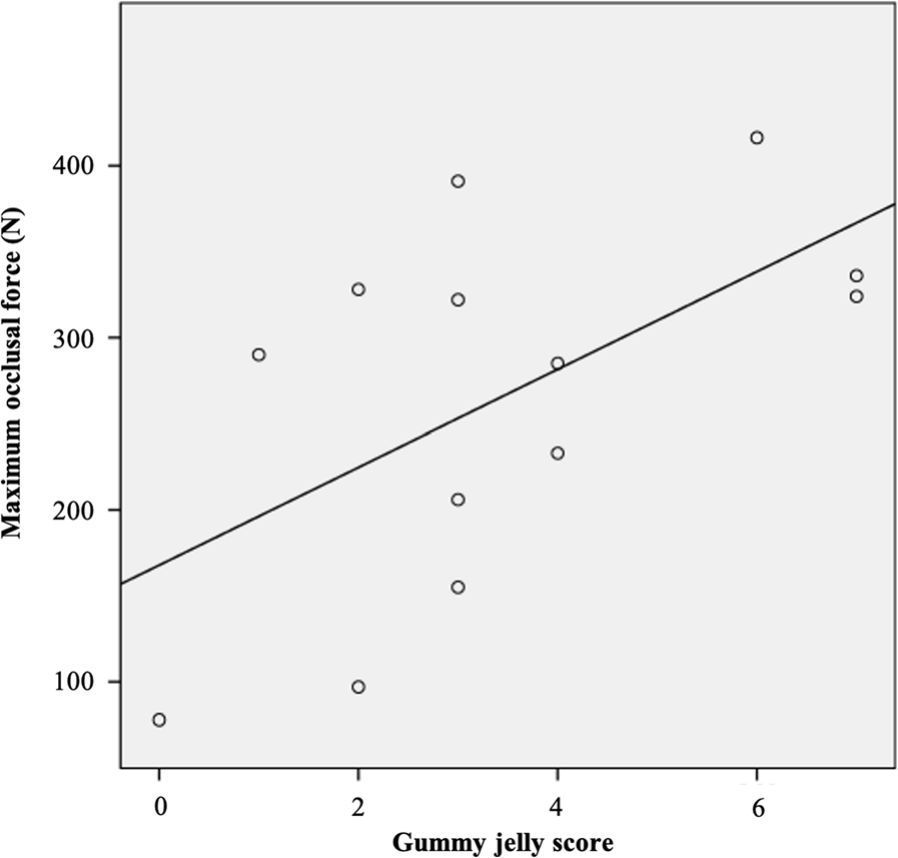
The scatter diagram shows the relationship between maximum occlusal force and gummy jelly. Significant positive correlation was observed (*r* = 0.537, *P* = 0.025) in a 5-year follow-up period. Solid line represents regression line

A Pearson correlation test was carried out to evaluate the correlation of MP and maximum occlusal force, and the correlation was statistically significant. A statistically significantly positive correlation between the maximum occlusal force and MP, both with color-changeable chewing gum (*r* = 0.676, *P* = 0.007, Fig. 2) and gummy jelly (*r* = 0.537, *P* = 0.025, Fig. 3), was observed at the 5-year evaluation time point. The correlation coefficient (*r*) with color-changeable chewing gum tended to be larger than that with gummy jelly.

## Discussion

With this unblind, parallel, randomized, and controlled clinical trial, we aimed to provide more evidence on the MP outcome of two loading protocols using a magnetic attachment. The overall result revealed no significant differences in color-changeable chewing gum, gummy jelly, and maximum occlusal force between the IL and CL groups at any evaluation time point. However, the results of this study indicated a positive correlation between maximum occlusal force and MP at the 5-year follow-up.

The outcome variables used in this study were MP and maximum occlusal force. These factors are generally accepted to assess the masticatory function of participants. When comparing the median of color-changeable chewing gum, gummy jelly, and maximum occlusal force between the IL and CL groups, statistically significant differences were not observed at any time point. However, a trend toward a further increase in color-changeable chewing gum has been reported within both groups. No difference in MP between the IL and CL groups can, in part, be explained by the performance of the implant material utilized in the present study. In particular, the evolution of implant systems, designs, and surface properties has allowed for shortened healing times. It was expected that the IL group would exhibit significantly higher MP than CL group, whereas the current study was analyzed in long-term follow-up starting from 3 years. These may have contributed to the high bone-to-implant contact and enhanced bone deposition [36]. Moreover, there were no significant differences in ACP classification between the IL and CL groups, which might have affected the results of long-term observation. In addition, long-term follow-up may contribute to neuromuscular adaptation after implant-treatment and reported an increase in myodynamic and electromyography parameters approaching the values for normal dentate subjects [37]. Furthermore, the lack of a significant increase may be related to the small sample size of both groups.

However, factors that can evaluate the effect of the loading protocol are not only MP and maximum occlusal force. Several studies reported no significant differences between IL and CL groups at different follow-up time points in each study, such as marginal bone loss [16, 17, 21, 38], and clinical outcomes, including plaque, gingival score, probing depth, and implant stability [21, 38]. The present data are in agreement with previous studies, which reported that MP increased over time with the transition from CDs to IODs, independent of the loading protocol [27]. Schuster et al. [39] reported no significant differences in MP between the groups at the 1-year follow-up. Furthermore, the results from this current study present a new outcome for long-term follow-up of MP and maximum occlusal force to the author’s knowledge. However, similar to the short follow-up time point in this current study, another study with a short follow-up time point by Komagamine et al. [30] reported only one difference between CL and IL in the gummy jelly test after 6 months of implant placement. According to Giannakopoulos et al. [40], an improvement in the MP of the IL of 2-IODs wearers after 3 months of function was observed, irrespective of the retention system used.

In the current study, an increase in MP and maximum occlusal force was reported within each group. The present data are in agreement with previous studies [1, 2, 4, 7, 12], in regard to the fact that 2-IODs were higher for improvement in MP than the provision of CDs. The color-changeable chewing gum was significantly higher than the baseline at the 3-year evaluation time point for IL group. The color-changeable chewing gum is soft and relatively comfortable to chew and form a bolus, which may be suitable for participants with compromised MP [41]. The mixing ability of the overdentures might have been influenced by the abraded denture teeth and might be correlated with occlusal contact area than community ability [42]. On the other hand, there was no significant difference in gummy jelly within each group at any evaluation time point. The texture of gummy jelly is elastic and might be too hard or bulky for edentulous patients with impaired mastication. Furthermore, the denture causes high attrition of the anatomical form of denture teeth, muscle weakness, and frailty of the participant; thus, it is more difficult to bite gummy jelly than bite the color-changeable chewing gum. There was no significant difference in the maximum occlusal force within each group at any evaluation time point. However, after the insertion of 2-IODs compared to pre-implant insertion of CD, the maximum occlusal force was improved in both the groups compared to baseline, similarly to previous studies [2, 4, 40]. The improvement of MP in this study was still observed after direct implant treatment. Thus, implant treatment greatly improves oral function over a long period [2].

The results of this study indicated a positive correlation between the MP and the maximum occlusal force at the 5-year follow-up. According to Fontijn-Tekamp et al. [43], a significant correlation between the maximum occlusal force and chewing efficiency was reported. In addition, our results indicate that the positive correlation coefficient of maximum occlusal force was likely to be higher with the color-changeable chewing gum (*r* = 0.676, *P* = 0.007) than with gummy jelly (*r* = 0.537, *P* = 0.025). Yamada et al. [44] reported that the test gummy jelly was significantly correlated with occlusal force and occlusal contact area. Thus, it was suggested that community ability is more easily influenced by factors related to the teeth or masticatory muscles than by tongue and lip functions. However, MP is not explained by maximal occlusal force alone. It is known that motor functions of masticatory organs such as the tongue, lips, cheeks, and mandible deteriorate with age and influence MP. Further investigation will be needed in a future study.

A post hoc analysis indicated that the statistical power for detecting differences in MP between the two loading groups for the 5-year evaluation period was 0.15, with a large effect size of 0.57. However, the power statistic value was quite small, which was attributed to the small sample size. Thus, this work should be considered a preliminary study. In a future study, an increase in the sample size of each group to 50 participants would increase substantially the power statistic value to 0.80.

One of the limitations of the present study was the small sample size; although participants had a long follow-up period, a single participant could have a large influence on the study results. However, the change in oral masticatory function could have been demonstrated in a much larger group of participants. Thus, the investigation should be considered a preliminary study. Nonetheless, numerous confounding factors may have affected the long-term outcomes reported, such as inconsistency of the participants’ maxillary occlusion status, elevator muscles, oral motor function, food texture, and food amount for each masticatory cycle. These confounding factors should be considered in future RCTs.

## Conclusions

Based on the findings of this current study, the following conclusions were observed:

1. After the long-term observation of 2-IODs with magnetic attachment, no significant differences in MP and maximum occlusal force were observed between the IL and CL.
2. Compared to pre-implant insertion of CD, the MP and maximum occlusal force tended to improve, and the MP measured by color-changeable chewing gum significantly increased at 3 years.
3. At 5 years, the maximum occlusal force was significantly correlated with the MP, both with the color-changeable chewing gum and gummy jelly.

## Data Availability

The datasets used and analyzed during the current study are available from the corresponding author on reasonable request.

## Abbreviations

ACP: American College of Prosthodontists
CD: Complete denture
CL: Conventional loading
IL: Immediate loading
IOD: Implant overdenture
MP: Masticatory performance
RCT: Randomized clinical trial
TMDU: Tokyo Medical and Dental University
UMIN: University Hospital Medical Information Network

## References

[1] Elsyad MA, Hegazy SA, Hammouda NI, Al-Tonbary GY, Habib AA (2014). Chewing efficiency and electromyographic activity of masseter muscle with three designs of implant-supported mandibular overdentures. A cross-over study. Clin Oral Implants Res.

[2] Van Der Bilt A, Burgers M, Van Kampen F, Cune M (2010). Mandibular implant-supported overdentures and oral function. Clin Oral Implants Res.

[3] Gambareli FR, Serra MD, Pereira LJ, Gavião MB (2007). Influence of measurement technique, test food, teeth and muscle force interactions in masticatory performance. J Texture Stud.

[4] Van Kampen F, Van Der Bilt A, Cune M, Fontijn-Tekamp F, Bosman F (2004). Masticatory function with implant-supported overdentures. J Dent Res.

[5] Van Kampen F, Van Der Bilt A, Cune M, Bosman F (2002). The influence of various attachment types in mandibular implant-retained overdentures on maximum bite force and EMG. J Dent Res.

[6] Feine JS, Carlsson GE, Awad MA, Chehade A, Duncan WJ, Gizani S, Head T, Lund JP, MacEntee M, Mericske-Stern R, Mojon P, Morais J, Naert I, Payne AG, Penrod J, Stoker GT, Tawse-Smith A, Taylor TD, Thomason JM, Thomson WM, Wismeijer D (2002). The McGill consensus statement on overdentures. Mandibular two-implant overdentures as first choice standard of care for edentulous patients. Montreal, Quebec, May 24-25, 2002. Int J Oral Maxillofac Implants.

[7] Elsyad MA, Shawky AF (2017). Masticatory function with ball and resilient telescopic anchors of mandibular implant-retained overdentures: a crossover study. Quintessence Int.

[8] Elsyad M, Mahanna F, Elshahat M, Elshoukouki A (2016). Locators versus magnetic attachment effect on peri-implant tissue health of immediate loaded two implants retaining a mandibular overdenture: a 1-year randomised trial. J Oral Rehabil.

[9] Kimoto K, Garrett NR (2003). Effect of mandibular ridge height on masticatory performance with mandibular conventional and implant-assisted overdentures. Int J Oral Maxillofac.

[10] Bakke M, Holm B, Gotfredsen K (2002). Masticatory function and patient satisfaction with implant-supported mandibular overdentures: a prospective 5-year study. Int J Prosthodont.

[11] Pera P, Bassi F, Schierano G, Appendino P, Preti G (1998). Implant anchored complete mandibular denture: evaluation of masticatory efficiency, oral function and degree of satisfaction. J Oral Rehabil.

[12] Cardoso RG, MELO LA, Barbosa GA, Calderon PD, Germano AR, Mestriner Junior W (2016). Impact of mandibular conventional denture and overdenture on quality of life and masticatory efficiency. Braz Oral Res.

[13] Kim H-Y, Lee J-Y, Shin S-W, Bryant SR (2012). Attachment systems for mandibular implant overdentures: a systematic review. J Adv Prosthodont.

[14] Grover M, Vaidyanathan AK, Veeravalli PT (2014). OHRQoL, masticatory performance and crestal bone loss with single-implant, magnet-retained mandibular overdentures with conventional and shortened dental arch. Clin Oral Implants Res.

[15] Cristache CM, Muntianu LAS, Burlibasa M, Didilescu AC (2014). Five-year clinical trial using three attachment systems for implant overdentures. Clin Oral Implants Res.

[16] Helmy M-D, Alqutaibi AY, El-Ella A, Shawky A (2018). Effect of implant loading protocols on failure and marginal bone loss with unsplinted two-implant-supported mandibular overdentures: systematic review and meta-analysis. Int J Oral Maxillofac Surg.

[17] Schincaglia GP, Rubin S, Thacker S, Dhingra A, Trombelli L, Ioannidou E (2016). Marginal bone response around immediate-and delayed-loading implants supporting a locator-retained mandibular overdenture: a randomized controlled study. Int J Oral Maxillofac Implants.

[18] Alsabeeha N, Atieh M, Payne AG (2010). Loading protocols for mandibular implant overdentures: a systematic review with meta-analysis. Clin Implant Dent Relat Res.

[19] Roe P, Kan JY, Rungcharassaeng K, Lozada JL (2011). Immediate loading of unsplinted implants in the anterior mandible for overdentures: 3-year results. Int J Oral Maxillofac Implants.

[20] Liao K-Y, Kan JY, Rungcharassaeng K, Lozada JL, Herford AS, Goodacre CJ (2010). Immediate loading of two freestanding implants retaining a mandibular overdenture: 1-year pilot prospective study. Int J Oral Maxillofac Implants.

[21] Elsyad M, Elsaih E, Khairallah A (2014). Marginal bone resorption around immediate and delayed loaded implants supporting a locator-retained mandibular overdenture. A 1-year randomised controlled trial. J Oral Rehabil.

[22] Attard NJ, Zarb GA (2005). Immediate and early implant loading protocols: a literature review of clinical studies. J Prosthet Dent.

[23] Turkyilmaz I, Sennerby L, Tumer C, Yenigul M, Avci M (2006). Stability and marginal bone level measurements of unsplinted implants used for mandibular overdentures: a 1-year randomized prospective clinical study comparing early and conventional loading protocols. Clin Oral Implants Res.

[24] Lorenzoni M, Pertl C, Zhang K, Wegscheider WA (2003). In-patient comparison of immediately loaded and non-loaded implants within 6 months. Clin Oral Implants Res.

[25] Szmukler-Moncler S, Salama H, Reingewirtz Y, Dubruille J (1998). Timing of loading and effect of micromotion on bone–dental implant interface: review of experimental literature. J Biomed Mater Res.

[26] Hansson H, Albrektsson T, Branemark P-I (1983). Structural aspects of the interface between tissue and titanium implants. J Prosthet Dent.

[27] Passia N, Abou-Ayash S, Reissmann DR, Fritzer E, Kappel S, Konstantinidis I, Königsmarck V, Mundt T, Stiesch M, Wolfart S, Ali S, Kern M (2017). Single mandibular implant study (SMIS)− masticatory performance− results from a randomized clinical trial using two different loading protocols. J Dent.

[28] Romeo E, Chiapasco M, Lazza A, Casentini P, Ghisolfi M, Iorio M, Vogel G (2002). Implant-retained mandibular overdentures with ITI implants: a comparison of 2-year results between delayed and immediate loading. Clin Oral Implants Res.

[29] Gatti C, Haefliger W, Chiapasco M (2000). Implant-retained mandibular overdentures with immediate loading: a prospective study of ITI implants. Int J Oral Maxillofac Implants.

[30] Komagamine Y, Kanazawa M, Sato D, Minakuchi S (2019). A preliminary comparison of masticatory performances between immediately loaded and conventionally loaded mandibular two-implant overdentures with magnetic attachments. Clin Implant Dent Relat Res.

[31] McGarry TJ, Nimmo A, Skiba JF, Ahlstrom RH, Smith CR, Koumjian JH (1999). Classification system for complete edentulism. J Prosthodont.

[32] Sato D, Kanazawa M, Kim YK, Yokoyama S, Omura Y, Ozeki M, Minakuchi S, Kasugai S, Baba K (2016). Immediate loading of two freestanding implants placed by computer-guided flapless surgery supporting a mandibular overdenture with magnetic attachments. J Prosthodont Res.

[33] Hama Y, Kanazawa M, Minakuchi S, Uchida T, Sasaki Y (2014). Properties of a color-changeable chewing gum used to evaluate masticatory performance. J Prosthodont Res.

[34] Hama Y, Hosoda A, Komagamine Y, Gotoh S, Kubota C, Kanazawa M, Minakuchi S (2017). Masticatory performance-related factors in preschool children: establishing a method to assess masticatory performance in preschool children using colour-changeable chewing gum. J Oral Rehabil.

[35] Nokubi T, Yoshimuta Y, Nokubi F, Yasui S, Kusunoki C, Ono T, Maeda Y, Yokota K (2013). Validity and reliability of a visual scoring method for masticatory ability using test gummy jelly. Gerodontology.

[36] Brånemark PI, Hansson BO, Adell R, Breine U, Lindström J, Hallénet O (1977). Osseointegrated implants in the treatment of the edentulous jaw. Experience from a 10-year period. Scand J Plast Reconstr Surg Suppl.

[37] Heckmann S, Heussinger S, Linke J, Graef F, Pröschel P (2009). Improvement and long-term stability of neuromuscular adaptation in implant-supported overdentures. Clin Oral Implants Res.

[38] Elsyad MA, Al-Mahdy YF, Fouad MM (2012). Marginal bone loss adjacent to conventional and immediate loaded two implants supporting a ball-retained mandibular overdenture: a 3-year randomized clinical trial. Clin Oral Implants Res.

[39] Schuster AJ, Marcello-Machado RM, Bielemann AM, Possebon APR, Chagas Júnior OL, Faot F (2020). Immediate vs conventional loading of Facility-Equator system in mandibular overdenture wearers: 1-year RCT with clinical, biological, and functional evaluation. Clin Implant Dent Relat Res.

[40] Giannakopoulos NN, Corteville F, Kappel S, Rammelsberg P, Schindler HJ, Eberhard L (2017). Functional adaptation of the masticatory system to implant-supported mandibular overdentures. Clin Oral Implants Res.

[41] Van Der Bilt A, Mojet J, Tekamp F, Abbink J (2010). Comparing masticatory performance and mixing ability. J Oral Rehabil.

[42] Sugiura T, Fueki K, Igarashi Y (2009). Comparisons between a mixing ability test and masticatory performance tests using a brittle or an elastic test food. J Oral Rehabil.

[43] Fontijn-Tekamp F, Slagter A, Van Der Bilt A, Van’T Hof MA, Witter DJ, Kalk WJ (2000). Biting and chewing in overdentures, full dentures, and natural dentitions. J Dent Res.

[44] Yamada A, Kanazawa M, Komagamine Y, Minakuchi S (2015). Association between tongue and lip functions and masticatory performance in young dentate adults. J Oral Rehabil.

